# Morphological and Imaging Characterization of the Eye and Adnexa of the Lesser Anteater ( *Tamandua tetradactyla* )

**DOI:** 10.1111/ahe.70126

**Published:** 2026-05-14

**Authors:** Estela Larissa Silva dos Santos, Amanda Vieira Fernandes, Francisco de Assis Dórea Neto, Caterina Muramoto, Danielle Nascimento Silva, Ana Cláudia Raposo, Marcia Maria Dantas de Faria, Ricardo Diniz Guerra e Silva, Alessandra Estrela Lima, Érica Augusta dos Anjos Cerqueira da Silva, Arianne Pontes Oriá

**Affiliations:** ^1^ School of Veterinary Medicine and Zootechny Federal University of Bahia Salvador Brazil; ^2^ Doctor of Veterinary Medicine Salvador Brazil; ^3^ Faculty of Veterinary Medicine and Animal Science University of São Paulo São Paulo Brazil; ^4^ National Council for Scientific and Technological Development Salvador Brazil

**Keywords:** anteaters, comparative anatomy, orbital anatomy, veterinary ophthalmology, wild mammals, Xenarthra

## Abstract

The southern tamandua ( *Tamandua tetradactyla* ) is a species vulnerable to environmental disturbances, largely due to increasing overlap between its natural habitat and anthropogenically modified landscapes. Detailed anatomical knowledge of the eye and orbital structures, supported by imaging and histological analyses, is essential to improve clinical, surgical and conservation practices for wild mammals. This study provides a comprehensive anatomical description of the eyeball and its adnexa in the southern tamandua. Twelve cadaveric specimens were examined using topographic dissection, orbital exenteration, bone maceration, ocular ultrasonography, computed tomography and histological processing. The orbit was incomplete and shallow, formed by the frontal, lacrimal, parietal, temporal and sphenoid bones, and exhibited a pronounced lateral projection of the eye. The eyeball was relatively small in proportion to the skull, globoid in shape, slightly elongated along the anteroposterior axis, and characterized by a high corneoscleral ratio. The eyelids were well developed, bearing eyelashes on both margins. The third eyelid was triangular and supported by hyaline cartilage. The lacrimal gland was reduced in size, whereas the Harderian gland was voluminous, lobulated and ring‐shaped, extensively surrounding the eye and occupying a substantial portion of the orbital cavity. Distinctive features of the extraocular musculature included duplication of the dorsal rectus muscle and absence of the dorsal oblique muscle. Ultrasonography enabled visualization of intraocular structures, and computed tomography confirmed orbital dimensions. Histological analysis revealed tissue organization consistent with the general mammalian pattern, with no evidence of a tapetum lucidum. These findings expand anatomical knowledge of Xenarthra and provide relevant reference data for veterinary ophthalmology, comparative anatomy and surgery, and the conservation of wild mammals.

## 1 Introduction

The superorder Xenarthra are mammals with distinctive morphological specializations, as the presence of accessory articulations in the lumbar vertebrae (xenarthrous joints), which provide increased axial rigidity. Additional adaptations include specialized rostral and lingual structures that facilitate tongue protrusion, as well as modifications of the thoracic limbs associated with an insectivorous diet in anteaters (Camilo‐Alves and Mourão 2006; Cruz et al. 2012; Miranda et al. 2014; Rossi et al. 2013). The locomotor and feeding adaptations are well‐documented; however, structures related to sensory systems remain comparatively less explored within this superorder.

The southern tamandua ( *Tamandua tetradactyla* ), a medium‐sized myrmecophagid widely distributed throughout South America, is currently classified as ‘Least Concern’ by the International Union for Conservation of Nature (Miranda et al. 2014). Despite its broad geographic distribution, regional anthropogenic pressures, including habitat fragmentation and road‐related mortality, highlight the importance of anatomical data to support clinical management and diagnostic assessment of the species.

The ocular apparatus plays a crucial role in environmental perception and behavioural modulation. These structures compose a system directly exposed to both biotic and abiotic pressures from the external environment, making it particularly susceptible to structural and functional alterations (Raposo et al. 2020).

The ocular system is composed of the eyeball, eyelids, third eyelid, extraocular muscles and lacrimal glands, all protected by the orbit. Species‐specific anatomical descriptions are essential for establishing reference morphological parameters and for avoiding inappropriate extrapolations based on domestic species (Boyda‐Andrade et al. 2024; Lantyer‐Araujo et al. 2019; Oriá et al. 2013; Superina and Abba 2020).

Imaging modalities are complementary tools to conventional anatomical approaches. Ultrasonography enables dynamic evaluation of soft tissues and intraocular components (El‐Boghdadly et al. 2021), whereas computed tomography provides detailed visualization of orbital bony structures (Moore and Lamb 2007; Karatag et al. 2025). The integration of anatomical characterization, imaging and histological data enhances the structural and functional understanding of the ocular system and provides valuable support for studies in comparative anatomy and veterinary ophthalmology (Oriá et al. 2015; Sena 2020).

Morphological descriptions of the ocular system are available for some Xenarthrans, such as the two‐toed sloth ( *Choloepus didactylus* ) and representatives of Cingulata (Aldana Marcos and Affanni 2005; Aldana Marcos et al. 2002; Klećkowska‐Nawrot et al. 2023). However, for *Tamandua tetradactyla* , detailed structural characterization of the eyeball and its adnexa remains limited, restricting comparative interpretations and clinical applications (Araújo et al. 2017). The present study aimed to characterize the morphology of the eyeball and its adnexa in *Tamandua tetradactyla* by integrating macroscopic, imaging (ultrasonography and computed tomography) and histological analyses. This approach seeks to contribute to the comparative anatomical knowledge of Xenarthra and to provide reference data for veterinary ophthalmology applied to wild mammals.

## 2 Material and Methods

### 2.1 Animals

Twelve adult southern tamandua ( *Tamandua tetradactyla* ) cadavers of gender undefined were used in this study. The specimens had died from natural causes or road accidents and showed no gross compromise of bones or internal organs. All cadavers were donated to the Veterinary Anatomy Sector of the School of Veterinary Medicine and Animal Science, Federal University of Bahia. The sample size reflects the limited availability of specimens of this species and is consistent with previous anatomical studies in wild mammals.

Post‐mortem examination of the eyes and adnexa was performed to confirm the absence of macroscopic abnormalities. Of the 12 specimens, 10 were used for dissection and fixation, two for skull maceration, one for ultrasonography and one for computed tomography. The study was approved by the Institutional Committee for the Use of Animals (protocol no. 15/2022), the Biodiversity Authorization and Information System (SISBIO; no. 82780‐1) and the National System for the Management of Genetic Heritage and Associated Traditional Knowledge (SISGEN; no. A1F8C27).

To ensure adequate preservation of internal structures, 10 specimens were fixed in a 10% aqueous formaldehyde solution (Ambiental Química, Salvador, Brazil). Prior to immersion, perfusion with the same solution was performed in the medial and lateral regions of the palpebral conjunctiva (0.2 mL each) and in the posterior segment of the eye. Subsequently, the heads were immersed in an equivalent fixative solution at a volume 10 times greater than that of the specimens and maintained for a period of 30 days.

After the fixation period, topographic dissection and orbital exenteration were performed. A circumferential incision along the orbital margin allowed displacement and complete removal of the eye together with its adnexa. Subsequently, the remaining orbital structures were carefully dissected to enable individual identification of the extraocular muscles, orbital glands, eyelids and the third eyelid.

Thereafter, a sagittal incision of the skin was made from the frontal to the nasal region of the head to expose the retrobulbar structures and to confirm their topographical relationships. During dissection, morphometric measurements of the ocular and orbital structures were obtained using a digital calliper (Mitutoyo, São Paulo, Brazil), following the methodology described by Sarma (2006). All stages of the procedure were documented using a high‐resolution digital camera equipped with a macro lens and flash system (Nikon D7000, Nikon Inc., Tokyo, Japan), with standardized angle, lighting and capture distance.

Craniometric parameters of the orbital region were also assessed according to the following criteria:

1. Orbital vertical length: the perpendicular distance between the supraorbital and infraorbital margins of the orbit;
2. Orbital horizontal width: the horizontal distance between the rostral and caudal margins of the orbital rim;
3. Orbital index: orbital width/orbital length × 100;
4. Orbital depth: the distance between the optic foramen and the center of the orbital rim;
5. Orbital area: calculated using the formula 22/7*ab*, where *a* and *b* correspond to half of the orbital length and width, respectively.

Interorbital distance was assessed using the following measurements:

1. Rostral level: distance between the junctions of the frontolacrimal sutures on each side at the rostral margin of the orbit;
2. Middle level: distance between the supraorbital margins of the orbit on each side;
3. Caudal level: distance between the junctions of the zygomatic bones at the caudal margin of the orbit on each side;
4. Frontal length: distance from the tip of the zygomatic process of the frontal bone to the frontolacrimal sutures;
5. Lacrimal length: distance from the frontolacrimal sutures to the junction between the lacrimal and zygomatic bones.

Craniometric reference points followed the description proposed by Schimming and Silva (2013):

1. Inion: central point on the external occipital protuberance;
2. Bregma: junction of the right and left frontoparietal sutures in the median plane;
3. Nasion: junction of the right and left frontonasal sutures in the median plane;
4. Prosthion: most rostral point of the interfrontal suture;
5. Basion: midpoint of the ventral margin of the foramen magnum;
6. Euryon: the most lateral point of the neurocranium;
7. Zygion: the most lateral point of the zygomatic arch.

The natural maceration process was performed on two skulls to expose the osseous structures of the orbit. Initially, the soft tissues of the skull were manually removed, while preserving muscle attachment sites and bony boundaries. The specimens were subsequently maintained partially submerged in water and exposed to ambient conditions, allowing controlled decomposition of residual tissues through microbial activity. Throughout the process, the container was kept in a well‐ventilated area and protected from adverse environmental conditions, and the specimens were monitored until complete removal of soft tissues was achieved. After maceration, the skulls were rinsed with water only to remove debris and air‐dried at room temperature, ensuring preservation of the fine anatomical details of the orbital region.

### 2.2 Ultrasonography and Computed Tomography

One cadaver previously fixed in a 10% aqueous formaldehyde solution was submitted to imaging evaluation. However, only one eye was included in the morphometric analyses, as lens luxation was observed in the contralateral eye during image acquisition, preventing the collection of reliable images and measurements. This limitation was considered during data interpretation. Ocular ultrasonography was performed using a high‐frequency linear transducer (L8–18i; GE Logiq F6 R2, General Electric Healthcare, Boston, MA, USA). Images were obtained using the transcorneal technique, with scanning of the ocular globe in vertical and horizontal axial planes.

A fresh cadaver was submitted to computed tomography (CT) (GE Brivo CT325, General Electric, Boston, MA, USA). Images were reconstructed with bone and soft tissue algorithms and visualized using Horos medical image viewer software, with a slice thickness of 1 mm. Image analysis was performed using multiplanar reconstruction (MPR) in sagittal, transverse and dorsal planes, allowing morphometric assessment of the orbital cavity and eye.

Biometric measurements were obtained from horizontal image sections, with the transducer positioned at the center of the cornea or eyelid. Proper positioning was confirmed when the posterior wall of the eye and key intraocular reference structures, including the cornea, lens, orbital canal and interorbital distances, were clearly visualized.

### 2.3 Histological Analysis

Fragments of the ocular globe and orbital adnexa were fixed in 10% formaldehyde solution (Ambiental Química, Salvador, Brazil), processed using routine histological techniques, embedded in paraffin, sectioned at 5 μm using a rotary microtome and stained with haematoxylin and eosin (H&E), Masson's trichrome and Alcian Blue. The lacrimal and orbital glands, third eyelid, conjunctiva, eye, cornea, sclera, iris, ciliary body, lens and retina were evaluated. The histological sections were examined using a Nikon CiL light microscope coupled to a DS‐Ri2 digital camera (Nikon Instruments Inc., Tokyo, Japan), and digital images were acquired under standardized conditions.

### 2.4 Anatomical Terminology

Anatomical nomenclature followed the Nomina Anatomica Veterinaria (ICVGAN 2017).

### 2.5 Statistical Analysis

The Shapiro–Wilk test was applied to assess the normality of variables obtained from measurements of the eye and its adnexa. Statistical analyses were performed using GraphPad Prism software (version 7.04; GraphPad Software, San Diego, CA, USA), and the level of significance was set at 5% (*p* < 0.05). Comparisons between left and right eyes were conducted using Student's *t*‐test. Results are expressed as mean ± standard deviation, along with 95% confidence intervals. Correlations between variables were evaluated using Pearson's correlation coefficient (*r*).

## 3 Results

### 3.1 Skull and Orbit

Craniometric and orbital measurements were obtained to characterize overall dimensions and to assess their relationship with the average body size of the individuals. All specimens were adults, and no significant differences were observed in total body length among individuals (*p* = 0.6298), with a mean length of 88.9 ± 7.17 cm (range: 83–98 cm). Body length excluding the tail and tail length were measured separately, yielding mean values of 52.4 ± 4.12 cm (range: 45–58 cm) and 36.5 ± 3.72 cm (range: 31–42 cm), respectively.

Measurement data for the orbit, eye and adnexa showed normal distribution according to the Shapiro–Wilk test (*p* > 0.39). Furthermore, no significant differences were detected between the right and left eyes for any of the variables analysed, as assessed by Student's *t*‐test for paired samples (*p* ≥ 0.21).

**FIGURE 1:**
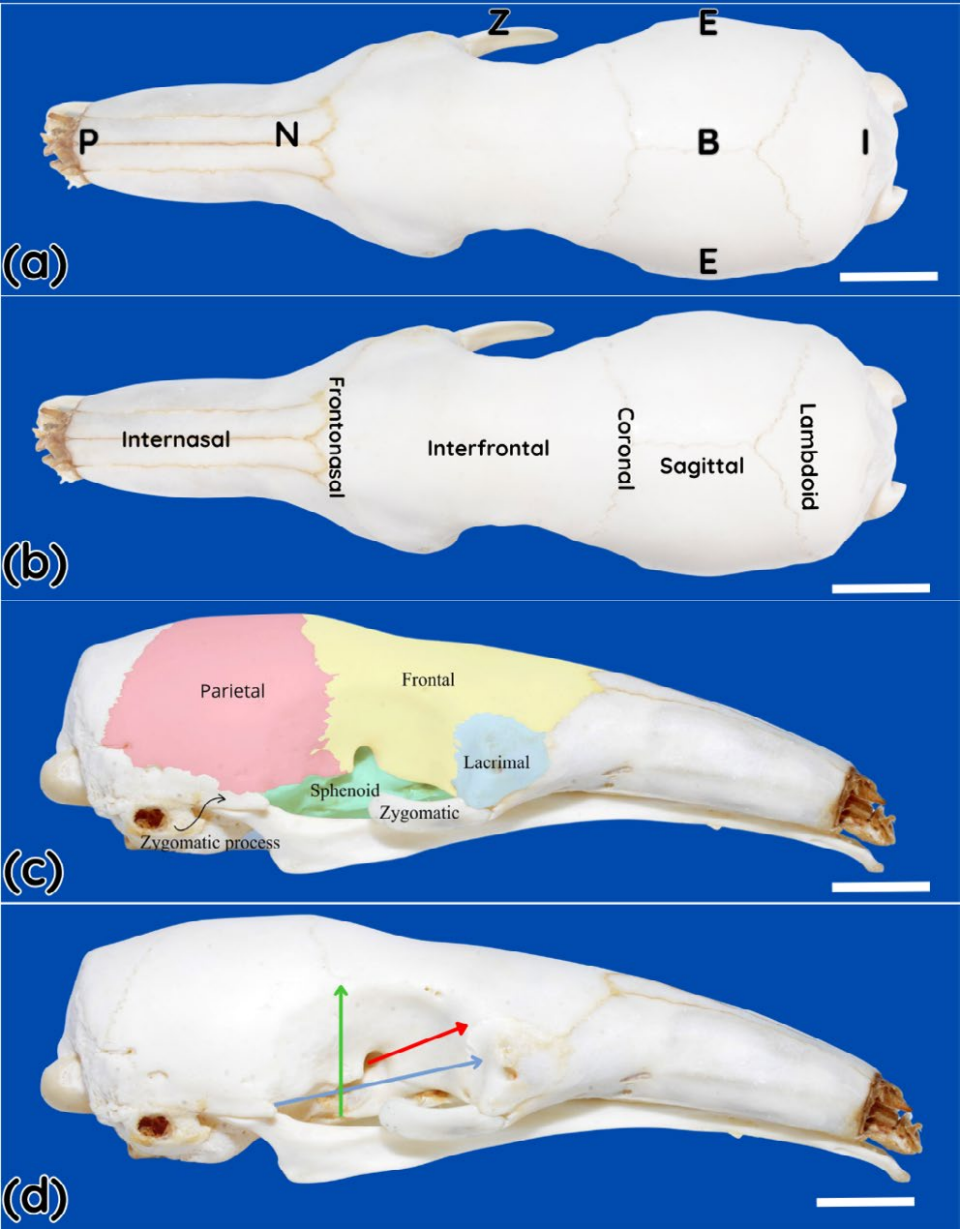
Osseous composition of the skull of the southern tamandua ( *Tamandua tetradactyla* ). (a) Dorsal view showing the main craniometric landmarks. The following reference points are indicated: B, bregma; E, euryon; I, inion; N, nasion; P, prosthion; R, rhinion; Z, zygion. (b) Cranial sutures observed in dorsal view. (c) Right lateral view of the orbit, highlighting the bones that compose it: blue, lacrimal bone; yellow, frontal bone; orange, parietal bone; pink, temporal bone; green, sphenoid bone; the zygomatic bone and its process are indicated by a dark arrow. (d) Orbital view illustrating the morphometric measurements of height, width and depth. The vertical orbital length (blue line), horizontal orbital width (green line) and orbital depth (red line) are indicated. Scale Bar = 1 cm.

Craniometric analysis of the macerated skull allowed determination of the total cranial length between the prosthion and inion (from the nasal to the occipital bone), which measured 119.8 mm, as well as the distance to the lambdoid suture, which was 35.6 mm. Cranial width, defined as the distance between the zygion points corresponding to the maximum lateral projection of the zygomatic arches, measured 65.2 mm (Figure 1). Based on the mean body length of the specimens, the head represents approximately 13.5% of the total body length.

The orbit was characterized as incomplete and formed by the frontal, lacrimal, parietal, temporal, and sphenoid bones, the latter being reduced and continued rostrally by cartilaginous tissue. The internasal, frontonasal, interfrontal, coronal, sagittal and lambdoid sutures were identified as anatomical reference boundaries. Mean orbital depth measured 14.9 mm, horizontal width 28.5 mm and vertical height 18.4 mm. Interorbital distances measured 23.0 mm in the macerated skull, 29.1 mm at the level of the coronal suture and 32.0 mm at the level of the frontonasal suture. The distance between the lacrimal extremity and the nasofrontal suture was 27.3 mm.

### 3.2 Ocular Adnexa

**FIGURE 2:**
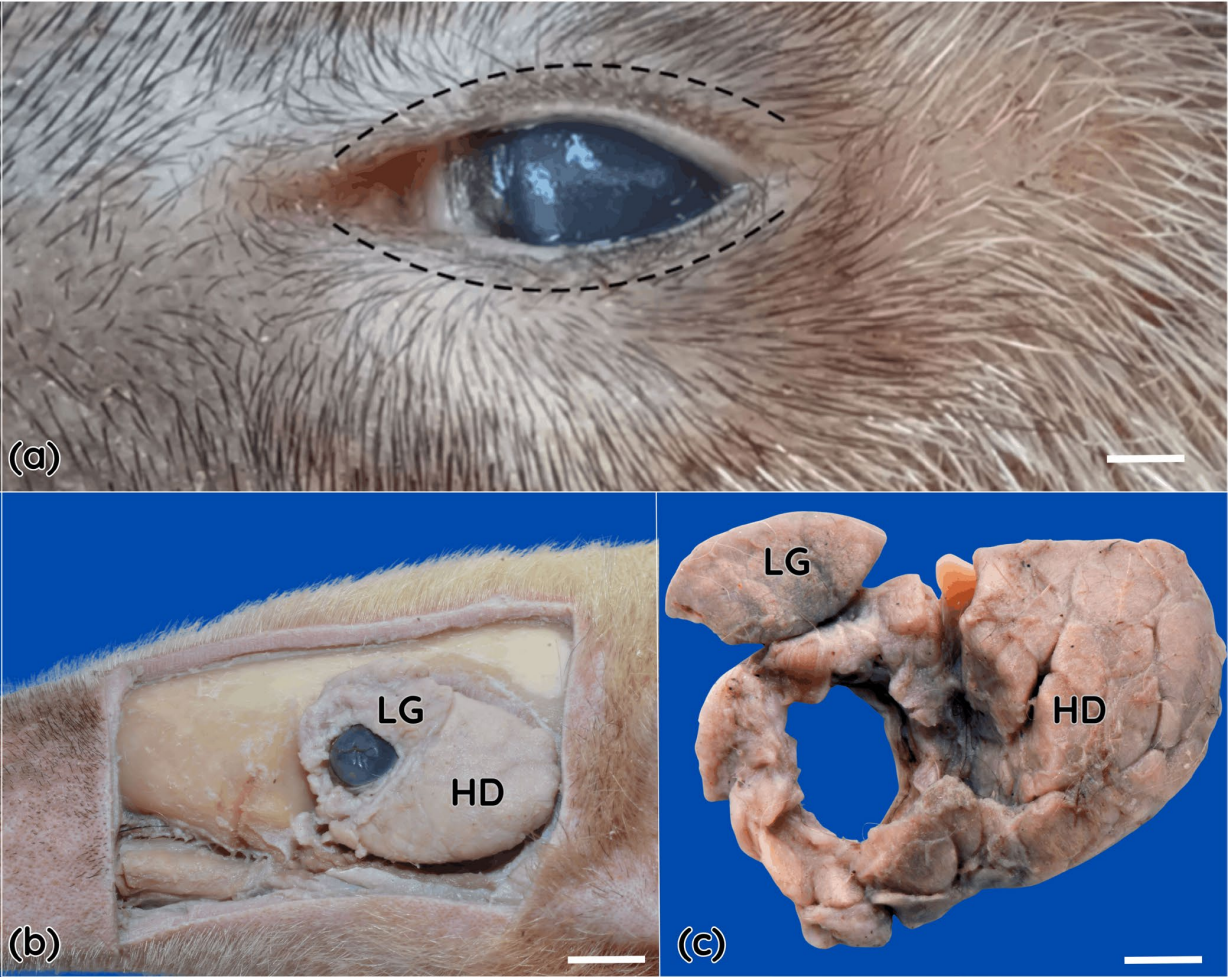
Macroscopic features of the ocular adnexa of the southern tamandua ( *Tamandua tetradactyla* ). (a) Eyelids showing ordinary hairs on the external surface and short eyelashes arranged along the free margins of both the upper and lower eyelids (dark dashed lines). The third eyelid is visible at the medial canthus, exhibiting a triangular shape and a pigmented margin. (b) Orbital glands of the southern tamandua after dissection in anatomical position. The lacrimal gland (LG) is smaller than the Harderian gland (HG). (c) Isolated appearance of the orbital glands after removal, demonstrating the lacrimal gland (LG) and the Harderian gland (HG). Scale Bar = 1 cm.

The eyelids of the specimens examined were well developed and completely covered the surface of the eye. The upper eyelid was thicker than the lower eyelid. Short eyelashes were observed along both the upper and lower palpebral margins, arranged in single rows (Figure 2). Openings of the tarsal glands were not observed.

The third eyelid was identified at the medial canthus and exhibited a triangular shape, a thickened base and an elongated cartilaginous plate, covered by conjunctiva and presenting a pigmented free margin. The palpebral conjunctival surface appeared smooth and well vascularized. The lacrimal gland was located dorsolateral to the eye, near the supraorbital margin, and was partially covered by connective tissue. It was small in size, oval to elongated in shape, whitish in colour, with a smooth external surface and firm consistency.

A structure consistent with the Harderian gland was observed. It was voluminous, lobulated and firm in appearance, extensively surrounding the eye in a medial and retrobulbar position. The gland occupied approximately 10.6% of the total orbital volume and exhibited an annular arrangement, forming a continuous structure that encircled the eye and extended into the ventral region of the orbit. Macroscopically, its surface displayed a yellowish to light brown coloration, with well‐defined lobules separated by clearly discernible connective tissue septa. The dimensions of the glands were similar between the eyes (Table 1).

**TABLE 1:** Mean, standard deviation (SD), minimum and maximum values of post‐mortem biometric measurements of 10 southern tamandua ( *Tamandua tetradactyla* ) specimens and their eyes and ocular adnexa.

Structure	Mean ± SD (mm)	Min–Max (mm)
Palpebral fissure	9.7 ± 1.70	8.2–13.7
Vertical eyeball diameter	7.0 ± 0.56	6.0–7.9
Horizontal eyeball diameter	7.0 ± 0.41	6.2–7.7
Vertical corneal diameter	6.2 ± 0.34	5.4–6.5
Horizontal corneal diameter	6.2 ± 0.42	5.7–6.7
Lacrimal gland length	9.1 ± 2.02	5.9–11.7
Lacrimal gland width	4.7 ± 1.48	3.2–5.3
Harderian gland length	19.0 ± 5.04	10.3–28.8
Harderian gland thickness	3.5 ± 1.66	1.9–6.5
Harderian gland height	12.5 ± 4.70	7.6–16.3

### 3.3 Eyeball

**FIGURE 3:**
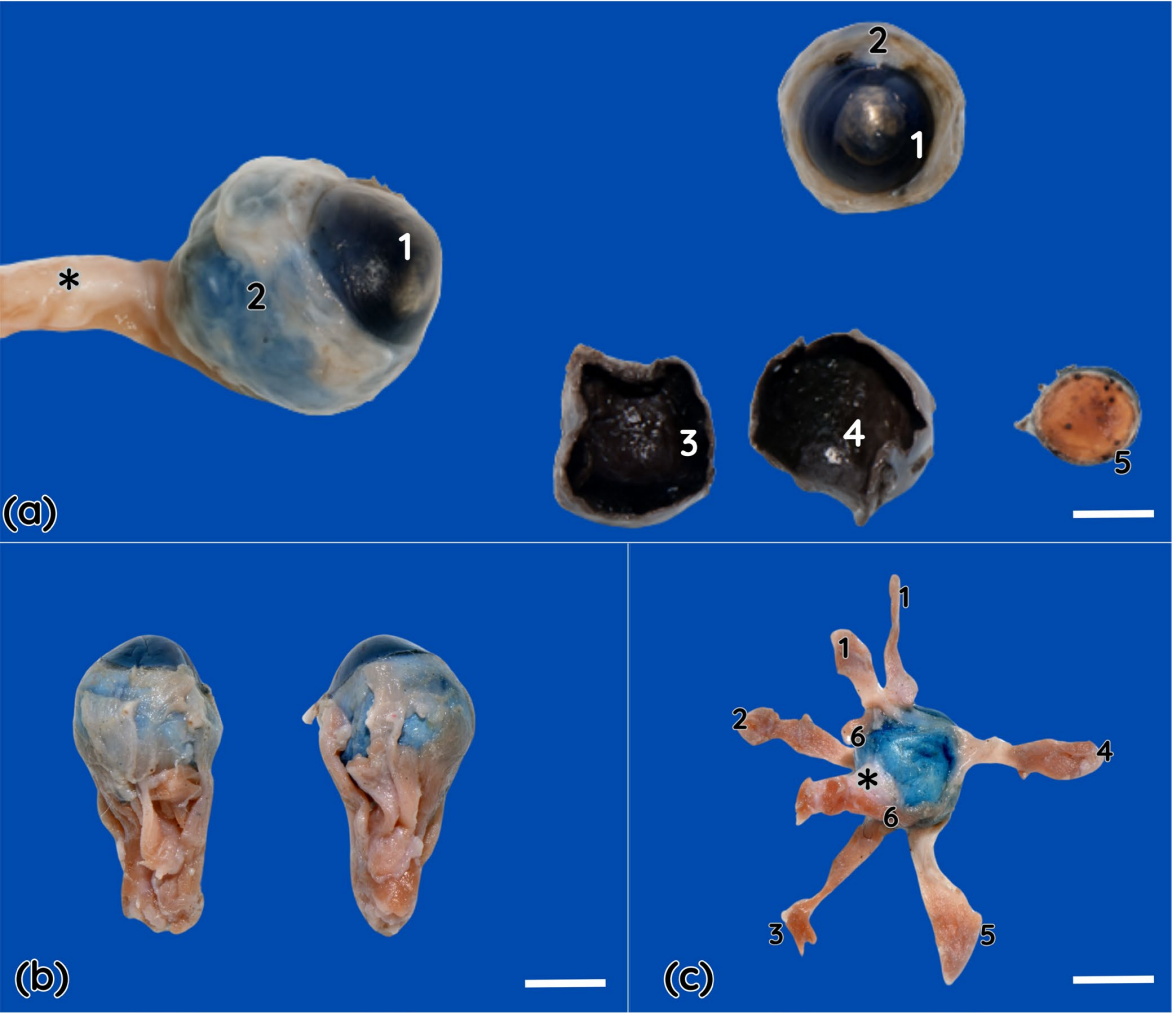
Ocular globe of the southern tamandua ( *Tamandua tetradactyla* ). (a) Lateral view of the ocular globe and adnexa, highlighting the paracornea (1) and sclera (2). A transverse section of the ocular globe demonstrates the anterior and posterior segments and the main internal structures, in which the vascular tunic is represented by the choroid, with no clear distinction of the retina (3 and 4). The lens (5) exhibits a biconvex shape. (b) The ocular globe is small and spherical, with visible insertions of the extraocular muscles. (c) Extraocular muscles are shown, including the rectus muscles: dorsal (1), medial (2), ventral (3) and lateral (5), the ventral oblique muscle (4) and the retractor bulbi muscle (6). Scale Bar = 1 cm. * optic nerve.

The eyeball was small in proportion to the size of the skull and exhibited a globoid shape, slightly elongated along the anteroposterior axis (Figure 3). It was positioned within a shallow and open orbit, projected laterally relative to the head profile, and occupied approximately 38% of the height of the bony orbital framework. The sclera was thick, whitish and opaque, covering most of the external surface of the eye. The cornea was transparent, slightly convex and well delineated, with a relative corneoscleral index corresponding to approximately 88% of the eyeball diameter along both axes. The iris was visible through the cornea as a circular, pigmented structure delimiting the central pupillary aperture. The lens was well developed, biconvex in shape, transparent and firmly adherent to its capsule.

Correlation analyses were performed using biometric data obtained from 10 fixed heads to identify potential proportional relationships among anatomical structures. A moderate and significant correlation was observed between the horizontal and vertical dimensions of the head (*r* = 0.666; *p* = 0.035), as well as between palpebral fissure length and corneal height (*r* = 0.500; *p* = 0.027). The Harderian gland showed a moderate negative correlation with the horizontal diameter of the eyeball, including gland height (*r* = −0.463; *p* = 0.040), thickness (*r* = −0.446; *p* = 0.049) and length (*r* = −0.481; *p* = 0.032).

### 3.4 Extraocular Muscles

The extraocular muscles inserted directly into the sclera and followed straight courses from their orbital origins. The medial, lateral and ventral rectus muscles were identified, as well as a paired dorsal rectus muscle (double dorsal rectus), in addition to the ventral oblique muscle and the retractor bulbi muscle.

The paired dorsal rectus consisted of two distinct and parallel muscular portions inserting on the dorsal surface of the eye. The dorsal oblique muscle and its trochlea were not identified. The retractor bulbi muscle was slender and composed of muscle bundles arranged around the optic nerve, forming a muscular cone in the retrobulbar region.

### 3.5 Ultrasound

**FIGURE 4:**
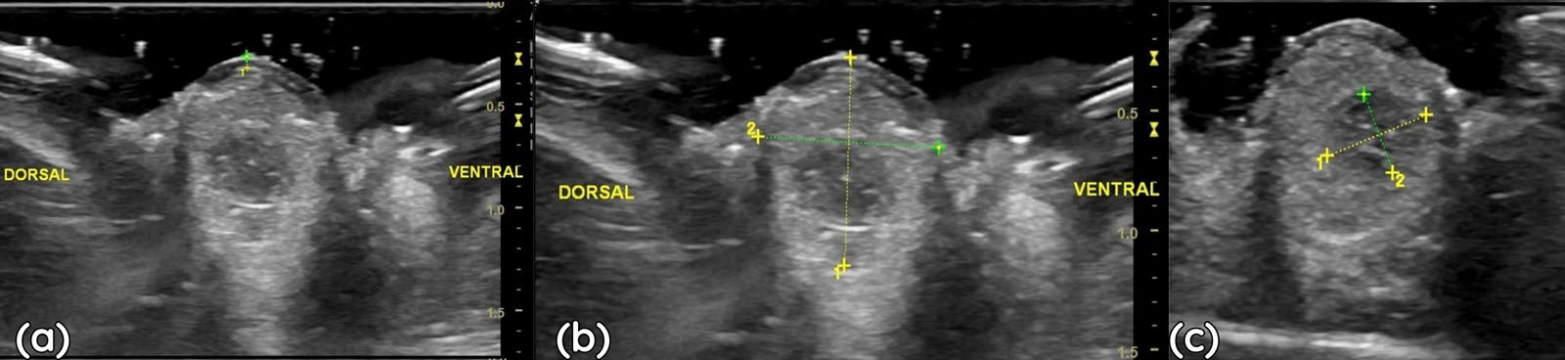
Ultrasonographic features of the ocular globe of the southern tamandua ( *Tamandua tetradactyla* ) in transverse sections. (a) Lines indicate the limits of corneal thickness. (b) Measurements of the dorsoventral and mediolateral axes of the ocular globe. (c) Measurements of the dorsoventral and mediolateral axes of the lens.

The eye exhibited a globoid shape, with clear delineation of its structures along the anteroposterior axis. The cornea was identified as an anterior hyperechoic line with a thickness of 0.8 mm. The anterior chamber appeared anechoic, with a depth of 1.8 mm. The lens was biconvex, presenting a hyperechoic capsule and a homogeneous anechoic interior, measuring 6.2 mm along the anteroposterior axis. The vitreous body was anechoic and homogeneous, filling the entire posterior cavity. The axial length of the eye was 14.2 mm, the dorsoventral diameter was 12.9 mm and the mediolateral diameter was 13.1 mm (Figure 4).

### 3.6 Computed Tomography

**FIGURE 5:**
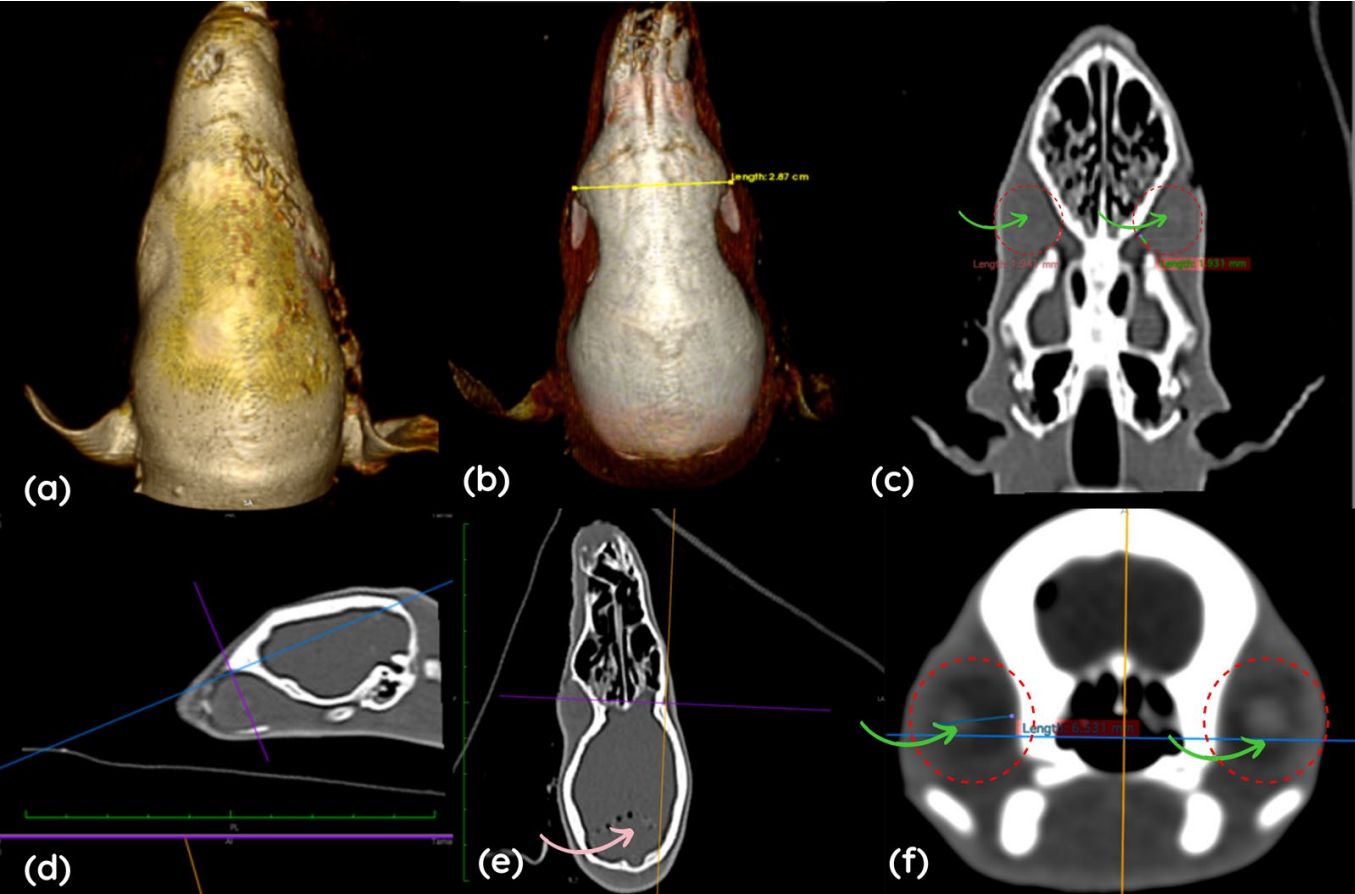
Computed tomography of the eye and ocular adnexa of the southern tamandua ( *Tamandua tetradactyla* ). (a) Three‐dimensional reconstruction in dorsal view. (b) Three‐dimensional dorsal reconstruction with soft tissue suppression, highlighting the interorbital distance between the frontal bones (yellow line). (c) Non‐contrast bone window with multiplanar reconstruction in dorsal view, demonstrating the orbital canal (green arrow), the ocular globe (red dashed circle) and the lens (green arrow). (d) Right orbital height between the frontal and zygomatic bones is indicated (blue and purple lines). (e) The brain shows multiple areas containing gas (pink arrow). (f) Transverse section in a non‐contrast bone window, in which the outline of the ocular globe is poorly defined (red dashed circle); the intraocular contents appear hypoattenuating, and the lens is slightly more hyperattenuating (green arrow). The blue line represents the mediolateral width measured in the sagittal plane.

Computed tomography enabled three‐dimensional evaluation of the orbit, eye, and lens in two eyes of *Tamandua tetradactyla* . The absence of orbital adipose tissue reduced tissue contrast and limited differentiation among the fibrous, vascular and neural tunics. Images acquired using the bone window clearly delineated the orbital boundaries and their dimensions (Figure 5).

The orbits were incomplete and continuous with the temporal fossa. In the left orbit, the rostrocaudal length measured 20.7 mm, height 12.7 mm and depth 9.2 mm. In the right orbit, length measured 24.0 mm, height 12.0 mm and depth 9.55 mm. Interorbital distance varied according to the reference point, measuring 25.0 mm between the frontal bones, 29.0 mm between the lacrimal bones and 21.0 mm at the medial frontal region. The orbital canal exhibited the same width in both eyes (2.0 mm) and a height of 1.3 mm.

The eye was poorly defined, and the intraocular contents appeared hypoattenuating, with the lenses positioned centrally. Measurements obtained using the soft tissue window revealed only minor variations between antimeres. Dorsoventral dimensions ranged from 7.6 to 7.8 mm in the left eye and from 7.4 to 7.8 mm in the right eye. Along the mediolateral axis (sagittal plane), values ranged from 6.3 to 6.7 mm on the left and from 6.4 to 6.5 mm on the right. In the dorsal plane, measurements along the same axis varied between 6.3 and 6.5 mm in both eyes.

With respect to the rostrocaudal axis (sagittal plane), the left eye exhibited measurements ranging from 8.0 to 8.3 mm, whereas the right eye ranged from 7.4 to 7.9 mm. In the dorsal plane, this axis showed values between 7.8 and 8.0 mm on the left and between 7.4 and 7.9 mm on the right.

Although the lenses were slightly more discernible, their margins were poorly defined, which hindered precise anatomical delineation. The dorsoventral diameter ranged from 3.4 to 3.7 mm, the mediolateral diameter was approximately 3.1 mm, and the rostrocaudal diameter ranged from 2.9 to 3.1 mm.

### 3.7 Histological Analysis

**FIGURE 6:**
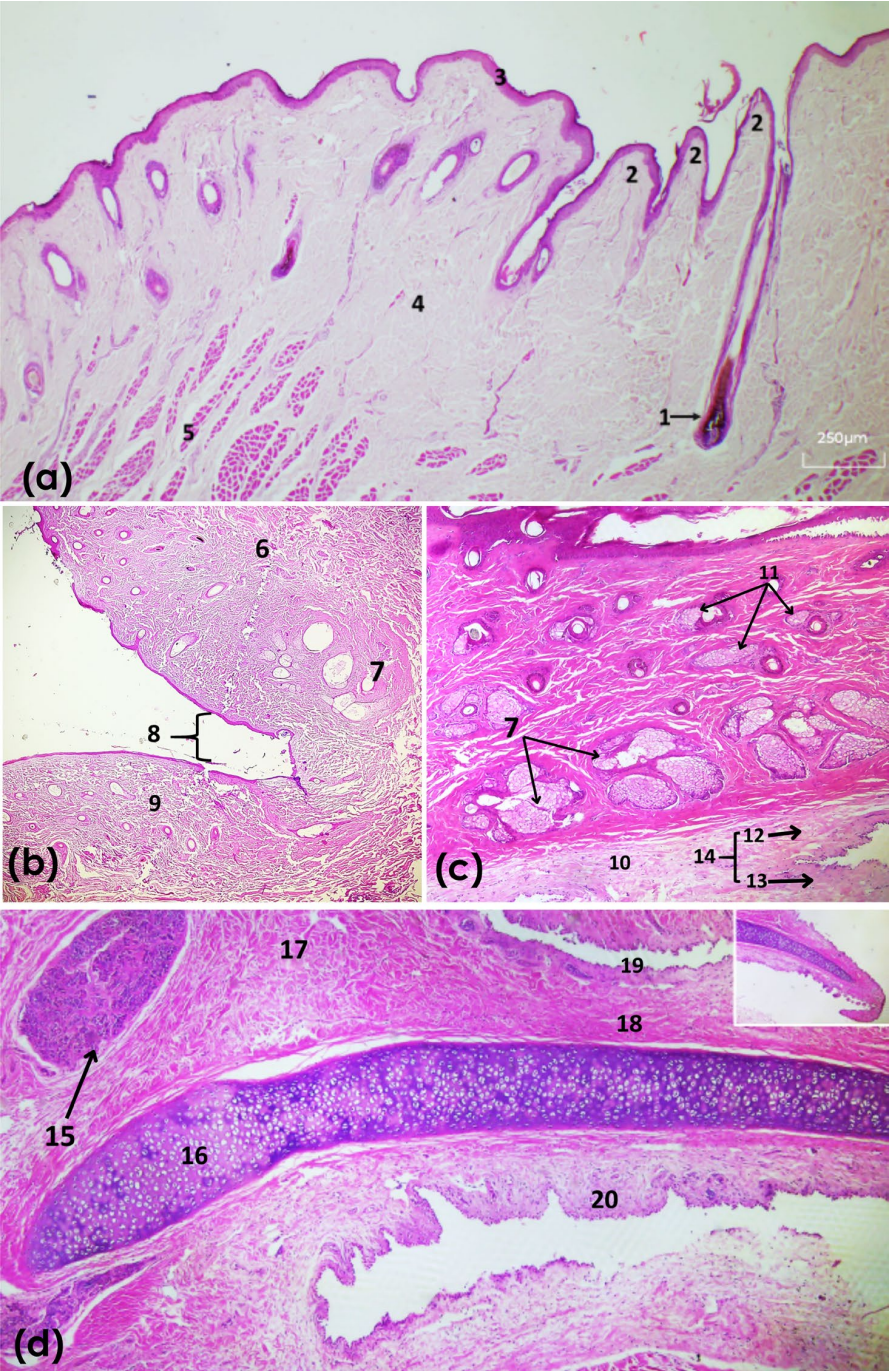
Photomicrographs of the adnexa of the southern tamandua ( *Tamandua tetradactyla* ) stained with haematoxylin and eosin (H&E). (a) Upper eyelid: (1) hair follicle; (2) epidermal folds; (3) epidermis; (4) dermis; (5) orbicularis oculi muscle. (b) Upper and lower eyelids (H&E, objective ×4): (6) upper eyelid; (7) tarsal (Meibomian) glands; (8) palpebral fissure; (9) lower eyelid. (c) Eyelid (H&E, objective ×4): (10) palpebral conjunctiva; (11) perifollicular glands (Zeis); (12) marginal zone; (13) bulbar zone; (14) fornix. (d) Third eyelid (H&E, objective ×4): (15) gland of the third eyelid; (16) cartilage; (17) Harderian gland; (18) connective tissue; (19) palpebral surface; (20) bulbar surface; (21) border of the third eyelid.

Histological sections of the upper and lower eyelids of the lesser anteater show an outer surface covered with skin and an inner surface lined by a mucous membrane. The epidermis consists of a stratified keratinized epithelium with five to six cell layers; the outermost is the stratum corneum and the innermost is the basal layer. In some regions, folds, epidermal papillae, are observed in this epidermis. The dermis is composed mostly of dense irregular connective tissue with hair follicles containing perifollicular glands, called Zeis glands. Adjacent to this portion of the dermis are striated skeletal muscle fibres, mostly arranged transversely, identified as the orbicularis oculi muscle (palpebral part), and a smaller number of longitudinally arranged muscles, such as the levator palpebrae superioris muscle. The innermost layer of the eyelid contains the tarsal or Meibomian glands. Near the tarsus, a thin layer of epithelial lining of the palpebral conjunctiva is observed, composed of pseudostratified epithelium, blood vessels and lymphatics. The conjunctiva covers the eyelid from its margin (marginal zone) to the bulbar portion (orbital zone), forming a fornix that covers the bulbar surface until it inserts into the limbus. The upper and lower eyelids join at the lateral and medial canthus to form the palpebral fissure or rima palpebral (Figure 6).

In the histological evaluation of the third eyelid, a thick layer of dense vascularized connective tissue was observed, harbouring hyaline cartilage with numerous chondrocytes and a small amount of intercellular substance. The chondrocytes are oval in shape and are mostly simple or form groups composed of 2 or 3 cells. The cartilage has a comma shape, being thick at the base and thin in the outer portion. It has two surfaces, a palpebral and a bulbar surface, both covered by epithelial cells. Surrounding the base of the cartilaginous tissue are located the glands of the third eyelid.

The gland of the third eyelid is divided into irregular lobules separated by bundles of reticular connective tissue, blood vessels and nerve fibres. They have a tubuloalveolar structure, forming acini that have cone‐shaped cells with granular cytoplasm and a rounded nucleus located near the base of the cells.

**FIGURE 7:**
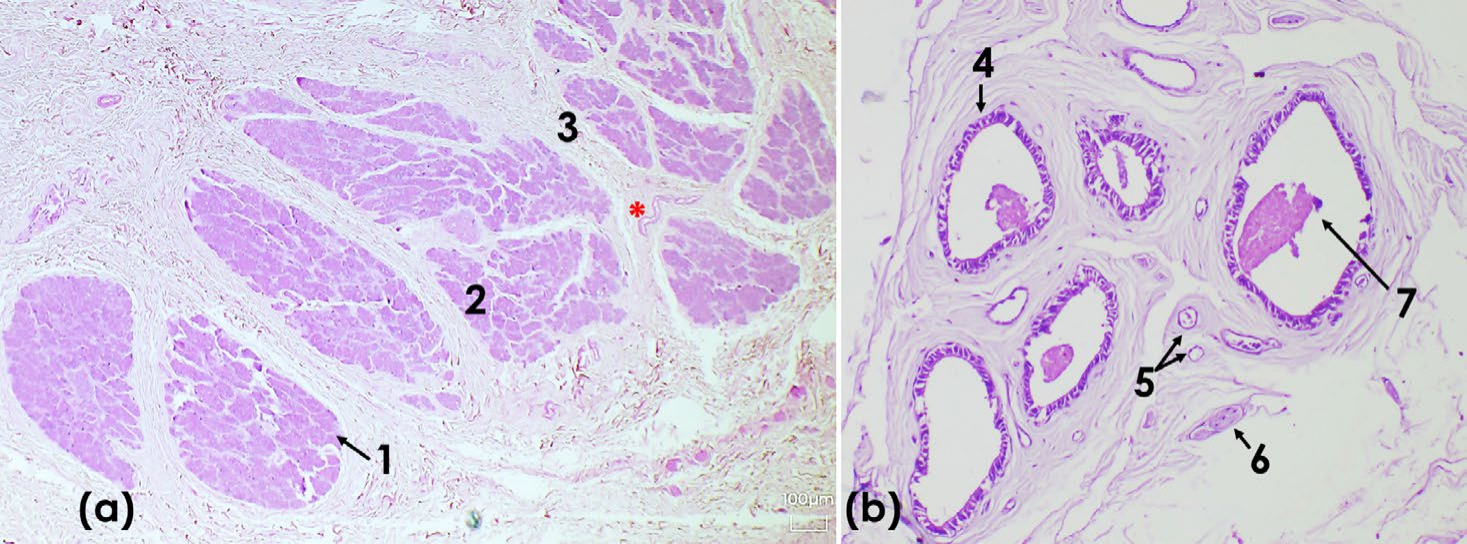
Photomicrographs of the adnexa of the southern tamandua ( *Tamandua tetradactyla* ) stained with haematoxylin and eosin (H&E). (a) Lacrimal gland (H&E, objective ×4). (1) serous acinus; (2) lobules; (3) connective tissue; (*) blood vessel. (b) Tubuloalveolar glandular parenchyma organized in lobules separated by connective tissue (H&E, objective ×10): (4) tubular alveolus; (5) capillaries; (6) nerve fibre bundle; (7) intraluminal secretion.

Histological sections of the lacrimal gland show a multilobar tubuloacinar gland with a predominance of acini over tubules. It was covered by a thick capsule of dense connective tissue that penetrated the glandular parenchyma, forming septa that separated it into small and large lobules. These lobules are composed of numerous serous acini, which have round nuclei located at the base of these cells. Blood vessels are also observed in the interstitium. The Harderian gland revealed multilobar tubuloalveolar glandular tissue with a predominance of alveoli, covered by a thick capsule of dense connective tissue with blood vessels. The connective tissue penetrated from the capsule into the glandular tissue, forming numerous thick and thin sparse septa that divided the gland into small and large lobules. The glandular parenchyma consists of epithelial cells lining the alveolar walls, forming tubules, with the lumen filled with amorphous and eosinophilic material. Between the lobes, bundles of nerve fibres and some adjacent smooth muscle fibres are observed (Figure 7).

The cornea has four layers: epithelium, membrane, stroma or substantia propria, Descemet's membrane and corneal endothelium. The anterior corneal epithelium is of the stratified squamous non‐keratinized type, consisting of five to six layers of epithelial cells, an inner layer formed by basal cells, two to three layers of polyhedral cells, and externally two layers of squamous cells. The basement membrane is attached to the basal cells. The stroma is formed by multiple layers of collagen fibres arranged in parallel and fibroblasts (keratinocytes), which are located between the collagen layers. Descemet's membrane delimits the stroma in the posterior portion of the cornea; it is also formed by collagen fibres. Between the cornea and the sclera, the limbus is observed, formed by a layer of pigmented basal cells; the stroma is vascularized and lacks pigmentation; and adjacent to this is the bulbar conjunctiva, formed by a stratified columnar epithelium. The sclera is formed by fibrous connective tissue organized in parallel and interwoven and is vascularized. Alcian blue highlighted the layers of the cornea, especially the epithelial layer.

**FIGURE 8:**
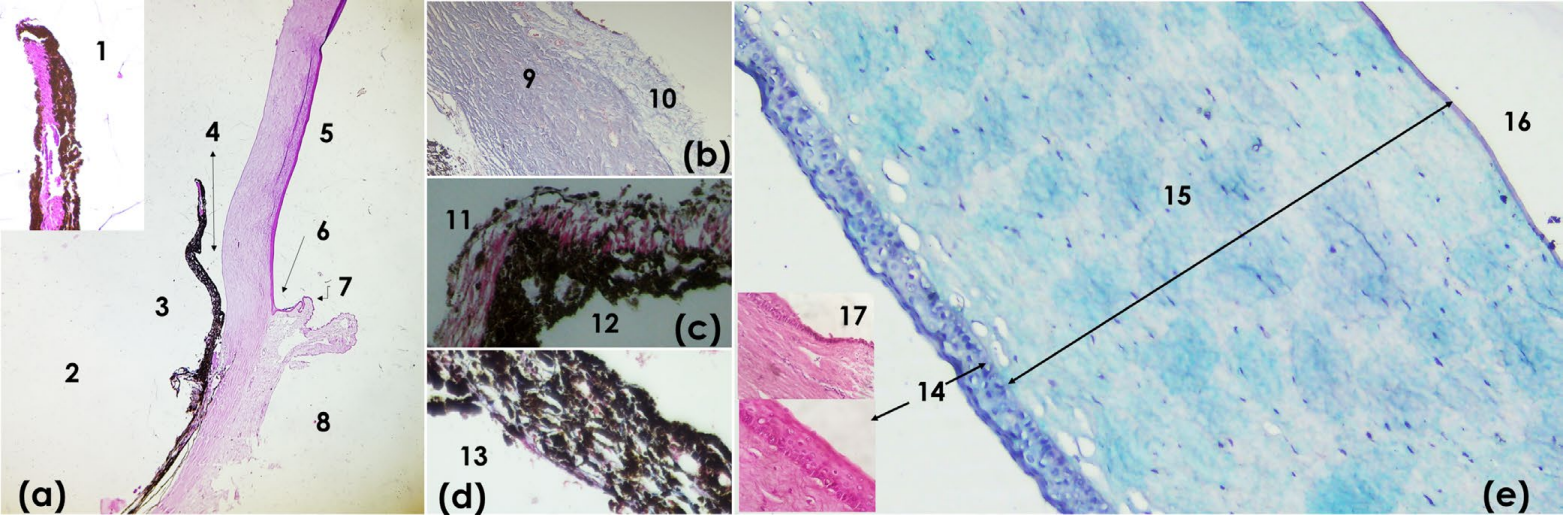
Photomicrographs of the ocular globe of the southern tamandua ( *Tamandua tetradactyla* ) stained with haematoxylin and eosin (H&E); Masson's trichrome and Alcian Blue. (a) Cornea, iris and adjacent structures: (1) iris sphincter muscle; (2) posterior chamber; (3) iris; (4) iridocorneal angle; (5) cornea; (6) limbus; (7) bulbar conjunctiva; (8) sclera (H&E obj. 4×). (b) Outer layer of the eye (Masson's trichrome obj. 10×): (9) sclera; (10) bulbar conjunctiva. (c) Iris (Masson's trichrome obj. 40×): (11) posterior epithelium; (12) anterior pigmented epithelium. (d) Choroide (13—Masson's trichrome obj. 40×). (e) Cornea (H&E and Alcian Blue obj. 10× and 40×): (14) epithelium; (15) stroma; (16) Descemet's membrane; (17) limbus.

Internally, the eyeball is divided into anterior chamber, posterior chamber and the vitreous body. In the anterior chamber, between the cornea and the iris, the iridocorneal angle is observed. The iris is part of the uvea and is located in the cranial portion of this structure. It has an elongated and thin free edge, intensely pigmented, divided into: anterior epithelium or outer epithelial layer directed towards the cornea; posterior epithelium or inner epithelial layer facing the lens, with less pigmentation. These layers are separated by the vascularized stroma, which presents, in the peripheral portion of the free edge, the sphincter or constrictor muscle of the pupil. Due to the intense pigmentation, it was not possible to clearly visualize the dilator muscle of the pupil at the base of the pigmented layer. The iris is connected to the ciliary body, which has, along with the ciliary muscle, formed by bundles of smooth muscle fibres, and the ciliary processes inserted and directed towards the posterior chamber. The choroid layer is composed of blood vessels, loose connective tissue and melanocytes (Figure 8).

The crystalline lens consists of a predominantly eosinophilic structure, divided into the following layers: lens equator, lens capsule, lens epithelium and lamellar fibres.

## 4 Discussion

The integration of macroscopic and microscopic anatomical descriptions with imaging modalities applied to the eye and its adnexa in *Tamandua tetradactyla* enabled a detailed characterization of the species' ocular system. These findings contribute to a better understanding of morphofunctional adaptations associated with the species' behaviour and habitat, while also providing relevant information for veterinary ophthalmology, comparative surgery and conservation strategies. Furthermore, this study provides morphological data that expand the comparative anatomical knowledge within the superorder Xenarthra and establish clinically relevant reference parameters.

The orbit of the southern tamandua ( *Tamandua tetradactyla* ) was incomplete, a characteristic also reported in the three‐toed sloth ( *Bradypus variegatus* ) (Sena 2020). This morphological similarity reflects the conservation of the typical orbital pattern within the superorder Xenarthra, resulting from the absence of complete fusion between the zygomatic and frontal bones. Consequently, direct communication between the orbit and the temporal fossa is maintained. The orbital margin was shallow, and the orbit relatively poorly developed in depth, lacking prominent bony processes that could serve as attachment sites for ligamentous structures. Both features may be associated with the lateral projection of the eye, as observed in *Tamandua tetradactyla* .

The eyelids of the southern tamandua ( *Tamandua tetradactyla* ) were well developed and presented eyelashes, features similar to those described in the two‐toed sloth ( *Choloepus didactylus* ) (Klećkowska‐Nawrot et al. 2023) and distinct from the pattern observed in the common sloth ( *Bradypus variegatus* ), in which eyelashes are restricted to the upper eyelid (Braz et al. 2020). The presence of eyelashes on both eyelids may contribute to ocular protection in environments with greater exposure to particulate matter and potential trauma or may compensate for increased ocular exposure to environmental variations and external agents (Gelatt and Plummer 2022; Kirbaşoğlu et al. 2021). By revealing species‐specific anatomical features, these findings allow a broader understanding of the selective pressures acting on the visual system and its interaction with the environment.

The structural measurements obtained in this study establish reference parameters that may assist in the identification of ocular abnormalities in wild animals. In addition, the third eyelid (located at the medial canthus and characterized by a triangular shape) was also observed. This conformation is widely recognized among phylogenetically related mammals, such as sloths (*Bradypus* and *Choloepus*) and armadillos (Order Cingulata) (Aldana Marcos and Affanni 2005; Braz et al. 2020; Klećkowska‐Nawrot et al. 2023). The persistence and morphological consistency of this accessory eyelid in both arboreal and fossorial species may reflect its functional importance, suggesting a key morphofunctional adaptation for ocular protection. Functionally, the third eyelid acts as an effective mechanical barrier against particulate debris and traumatic environmental agents, thereby preserving the integrity of the corneal and conjunctival surfaces.

The morphological architecture of the Harderian gland in *Tamandua tetradactyla* , characterized by a peribulbar annular arrangement, represents a distinctive anatomical finding that differs from the patterns described in other Xenarthrans. In sloths and armadillos, this gland typically exhibits a predominantly triangular configuration and is located strictly medial to the eye (Weaker 1981; Aldana Marcos and Affanni 2005; Braz et al. 2020; Klećkowska‐Nawrot et al. 2023), whereas in the southern tamandua a circumferential expansion surrounding the eye was observed. Despite these topographical differences, the lobulated nature of the gland appears to be conserved among the species mentioned. The coexistence of a reduced lacrimal gland and a voluminous annular Harderian gland suggests a distinct morphostructural configuration within the Xenarthra group. Future functional studies addressing the contribution of these glands to tear film composition will be essential to elucidate the physiological and evolutionary adaptations of this species.

The orbital insertion occupies the entire space between the eye and the orbital wall, a region that in other species is predominantly filled with dense adipose tissue (Lantyer‐Araujo et al. 2019). This arrangement suggests that, in addition to synthesizing components of the tear film, the gland may also play a mechanical protective role for the eye, functioning as a cushioning and thermal insulating element in densely vegetated, humid habitats that are often characterized by high temperatures (Dadson et al. 2025).

In sloths, both classical descriptions (Wislocki 1928) and more recent studies (Braz et al. 2020; Klećkowska‐Nawrot et al. 2023) do not report duplication of the rectus muscles or the absence of the dorsal oblique muscle, indicating that these features represent morphological particularities of the southern tamandua ( *Tamandua tetradactyla* ) relative to other Xenarthrans. In general, the extraocular muscles were small and slender, which may limit the range of eyeball movement. The duplication of the dorsal rectus muscle may enhance ocular stabilization during head movements or substrate exploration, whereas the absence of the dorsal oblique muscle suggests a simplification of torsional control. These findings point to a possible functional reorganization of ocular motility and warrant further comparative investigation in other representatives of Xenarthra to determine whether these features represent clade specific.

In armadillos ( *Chaetophractus villosus* ), the deep positioning of the eye within the orbit has been interpreted as a protective adaptation associated with their fossorial lifestyle (Aldana Marcos and Affanni 2005). Similarly, in the southern tamandua ( *Tamandua tetradactyla* ), the complete orbital insertion may provide mechanical protection to the eye in densely vegetated environments, where branches and other natural elements may pose a greater risk to the ocular surface. The relatively small size of the ocular globe reflects the species' visual acuity and sensory demands, as larger eyes are generally associated with increased light capture and improved image resolution (Hall et al. 2012; Kemp and Kirk 2014). In the southern tamandua, the reduced eye volume, together with the previously described low visual acuity (Miranda et al. 2014), indicates a functional adaptation to limited reliance on vision. Nevertheless, the relatively large corneal proportion in relation to overall eye size may enhance light transmission and potentially improve visual efficiency (Winkler et al. 2015).

The lens was centrally positioned within the eye and exhibited a circular biconvex shape, consistent with descriptions reported for dogs and sloths (Braz et al. 2020; Gelatt and Plummer 2022). Zonular ligaments were not macroscopically observed, likely due to their extreme delicacy and susceptibility to alterations induced by fixation and freezing procedures, or possibly due to the limited affinity of zonular fibres for conventional histological stains (Bassnett 2019). The apparent rigidity of the lens likely reflects post‐mortem tissue changes and the preservation methods employed (Prieto‐Bonete et al. 2015). In addition, post‐mortem physicochemical alterations may modify lens elasticity, making it difficult to accurately assess its conformation and biomechanical properties.

The same factors may explain the absence of clear macroscopic differentiation between the retina and choroid in the examined southern tamandua specimens, a finding similar to that reported in post‐mortem studies of the three‐toed sloth ( *Bradypus variegatus* ) (Braz et al. 2020). Imaging examinations allow noninvasive evaluation of the anatomy of wild animals and therefore represent valuable tools for diagnosis and prognosis (Prasad et al. 2021), while also corroborating the structural features described in the anatomical analysis.

Ocular ultrasonography in the southern tamandua ( *Tamandua tetradactyla* ) enabled the identification of the cornea, lens, anterior chamber and vitreous body, as well as the characterization of the overall proportions of the eye, in a manner comparable to studies conducted in *Bradypus variegatus* (Braz et al. 2020; Sena 2020) and *Choloepus didactylus* (Klećkowska‐Nawrot et al. 2023). Computed tomography confirmed the incomplete orbit and the reduced depth of the orbital cavity. Notably, there is a scarcity of scientific literature describing the application of this imaging modality to the ocular system of Xenarthra. These imaging techniques are particularly valuable for the diagnosis of ocular diseases, especially in situations where direct visualization of intraocular structures is not feasible.

The histological analysis revealed that the ocular structures exhibited an organization consistent with the pattern described for mammals. The lacrimal gland showed serous acini and a lobular architecture, whereas the Harderian gland displayed a well‐defined lobulated structure. In armadillos, both glands are described as lobular and secretory (Weaker 1981; Aldana Marcos and Affanni 2005; Aldana Marcos et al. 2002). Similarly to studies in sloths, the third eyelid contains a supporting hyaline cartilage covered by conjunctiva and non‐keratinized epithelium (Braz et al. 2020; Klećkowska‐Nawrot et al. 2023), a feature that has also been documented in armadillos (Aldana Marcos and Affanni 2005).

The cornea, sclera, iris, ciliary body, lens and retina exhibited a typical histological architecture, with well‐defined epithelial and stromal layers, organized fibres, and absence of a tapetum lucidum. These findings are consistent with descriptions reported for *Bradypus variegatus* (Braz et al. 2020) and *Choloepus didactylus* (Klećkowska‐Nawrot et al. 2023), reinforcing the histological similarity among representatives of the Xenarthra group.

The cornea displayed the characteristic five‐layer organization described in mammals, including a well‐defined epithelium, stroma and posterior limiting lamina (Descemet's membrane). However, no marked stromal thickening or cellular specializations suggestive of enhanced nocturnal adaptation were observed. In mammals with predominantly nocturnal habits, corneal thickness and rod density in the retina tend to be increased in order to maximize light capture (Hall et al. 2012; Kemp and Kirk 2014).

Although *Tamandua tetradactyla* is primarily nocturnal, the corneal architecture did not exhibit extreme morphological specializations, suggesting that visual acuity may not represent the species' primary sensory modality. The absence of a tapetum lucidum is consistent with previous descriptions in other Xenarthran species (Braz et al. 2020; Klećkowska‐Nawrot et al. 2023). In mammals, the presence of the tapetum lucidum is commonly associated with enhanced scotopic vision and nocturnal specialization (Gelatt and Plummer 2022). Its absence in *Tamandua tetradactyla* suggests a visual system that is not primarily adapted for extreme light amplification under low‐light conditions, reinforcing the hypothesis that olfactory and tactile cues play a more dominant ecological role in this species (Miranda et al. 2014).

In comparison with carnivores and primates, which exhibit marked ocular specialization associated with predatory behaviour and stereoscopic vision (Hall et al. 2012), *Tamandua tetradactyla* presents simplified extraocular musculature and a relatively reduced eyeball, features consistent with a lower reliance on visual acuity. The imaging findings corroborated the anatomical observations, particularly with respect to orbital incompleteness and the positioning of the eye, reinforcing the structural–functional interpretation related to ocular exposure and mechanical protection.

## 5 Conclusion

The integrated analysis of macroscopic anatomy, histology and imaging modalities enabled the characterization of distinctive features of the ocular system in *Tamandua tetradactyla* , including an incomplete orbit, a reduced and laterally positioned ocular globe, an annular Harderian gland, and duplication of the dorsal rectus muscle. Although the histological organization followed the general mammalian pattern, it was associated with structural adaptations consistent with a reduced reliance on vision. These findings expand the comparative anatomical knowledge within Xenarthra and provide relevant anatomical data for clinical practice and wildlife conservation.

## Author Contributions

A.P.O. and É.A.A.C.S. designed the experimental study and critically revised the manuscript. E.L.S.S., A.V.F., F.A.D.N. C.M. and D.N.S. executed the study. A.C.R., M.M.D.F., R.D.G.S. and A.E.L. contributed to the analysis of the overall experimental data used in this study. A.P.O., É.A.A.C.S., E.L.S.S. and A.C.R. participated in discussion. All authors contributed to drafting the manuscript and gave final approval of the version to be published.

## Funding

The authors have nothing to report.

## Ethics Statement

The study was approved by the Institutional Committee for the Use of Animals (protocol no. 15/2022), the Biodiversity Authorization and Information System (SISBIO; no. 82780‐1) and the National System for the Management of Genetic Heritage and Associated Traditional Knowledge (SISGEN; no. A1F8C27).

## Conflicts of Interest

The authors declare no conflicts of interest.

## Data Availability

The data that support the findings of this study are available from the corresponding author upon reasonable request.
